# Treatment and outcomes of anticoagulated geriatric trauma patients with traumatic intracranial hemorrhage after falls

**DOI:** 10.1007/s00068-022-01938-7

**Published:** 2022-03-10

**Authors:** Charlie J. Nederpelt, Leon Naar, Karien Meier, Suzanne F. M. van Wijck, Pieta Krijnen, George C. Velmahos, Haytham M. A. Kaafarani, Martin G. Rosenthal, Inger B. Schipper

**Affiliations:** 1 Department of Trauma Surgery, Leiden University Medical Center, Leiden, The Netherlands; 2 Division of Trauma, Emergency Surgery and Surgical Critical Care, Massachusetts General Hospital, Boston, MA United States; 3 Department of Trauma Surgery, Erasmus Medical Center, Rotterdam, The Netherlands

**Keywords:** Geriatric trauma, Neurotrauma, Direct oral anticoagulants, Vitamin K antagonists, Anticoagulant reversal

## Abstract

**Introduction:**

Emergency physicians and trauma surgeons are increasingly confronted with pre-injury direct oral anticoagulants (DOACs). The objective of this study was to assess if pre-injury DOACs, compared to vitamin K antagonists (VKA), or no oral anticoagulants is independently associated with differences in treatment, mortality and inpatient rehabilitation requirement.

**Methods:**

We performed a review of the prospectively maintained institutional trauma registry at an urban academic level 1 trauma center. We included all geriatric patients (aged ≥ 65 years) with tICH after a fall, admitted between January 2011 and December 2018. Multivariable logistic regression analysis controlling for demographics, comorbidities, vital signs, and tICH types were performed to identify the association between pre-injury anticoagulants and reversal agent use, neurosurgical interventions, inhospital mortality, 3-day mortality, and discharge to inpatient rehabilitation.

**Results:**

A total of 1453 tICH patients were included (52 DOAC, 376 VKA, 1025 control). DOAC use was independently associated with lower odds of receiving specific reversal agents [odds ratio (OR) 0.28, 95% confidence interval (CI) 0.15–0.54] than VKA patients. DOAC use was independently associated with requiring neurosurgical intervention (OR 3.14, 95% CI 1.36–7.28). VKA use, but not DOAC use, was independently associated with inhospital mortality, or discharge to hospice care (OR 1.62, 95% CI 1.15–2.27) compared to controls. VKA use was independently associated with higher odds of discharge to inpatient rehabilitation (OR 1.41, 95% CI 1.06–1.87) compared to controls.

**Conclusion:**

Despite the higher neurosurgical intervention rates, patients with pre-injury DOAC use were associated with comparable rates of mortality and discharge to inpatient rehabilitation as patients without anticoagulation exposure. Future research should focus on risk assessment and stratification of DOAC-exposed trauma patients.

## Introduction

Direct oral anticoagulants (DOACs) were first introduced in 2008 for the prevention of thromboembolism after elective arthroplasty, and in 2010 for non-valvular atrial fibrillation (AF) patients [1–5]. DOACs act either through direct thrombin inhibition (TDI, i.e. dabigatran) or through activated factor X inhibition (FXa, rivaroxaban, edoxaban, apixaban, betrixaban). The half-lives for DOACs are markedly shorter compared to those of vitamin K antagonists (VKA), which inhibit the vitamin-K dependent production of Factors II, VII, IX and X. In the United States, DOACs are currently the most prescribed oral anticoagulant for new indications of AF [6]. Physician and patient preference of DOACs over VKAs may be due to advantages including more predictable pharmacokinetics and pharmacodynamics, fewer interactions with other medication, and the fact that no laboratory monitoring is indicated with chronic use [7, 8].

At the same time, DOAC use may add considerable risks to the setting of acute major hemorrhaging. [9]. Emergency physicians and trauma surgeons are increasingly confronted with DOAC-associated injury and hemorrhage [10–13]. Those using oral anticoagulants (OAC), be it DOACs or VKAs, are typically frail, elderly patients with co-morbidities, co-medication and reduced mobility and stability. As a result, this population is at an increased risk of falls and accidents and associated traumatic injuries. Traumatic intracranial hemorrhage (tICH), complicated by anticoagulant medication, is of particular concern due to the potentially life-threatening consequences of small increases in hematoma volume within the cranial cavity.

Despite that DOAC use is prevalent in the geriatric trauma population, many aspects of the clinical presentation, management and prognosis are still unknown. This unfamiliarity potentially influences the treatment of tICH in DOAC-anticoagulated trauma patients. For instance, where reversal strategies for VKA-anticoagulated patients are widely established, reversal protocols for DOACs are still in the development phase, and based on less robust clinical evidence. Two DOAC reversal agents are currently available: idarucizumab to reverse dabigatran and andexanet alfa for reversal of factor Xa inhibitors, but clinical hemostatic effectiveness of both agents is yet to be confirmed in randomized controlled trials [14–16]. Furthermore, tICH patients may benefit from (decompressive) neurosurgical interventions, but a paucity of clinical data on neurosurgical exists and recent guidelines do not include specific recommendations on patient selection in the context of pre-injury DOAC exposure [17, 18].

The primary objective of this study was therefore to establish if pre-injury DOAC use in geriatric patients sustaining traumatic intracranial hemorrhage after falls is associated with differences in the rate of reversal agent use and of neurosurgical intervention, and with increased mortality and inpatient rehabilitation requirement.

## Methods

This study is reported on in accordance with the STrengthening the Reporting of OBservational studies in Epidemiology (STROBE) statement [19]. A retrospective review was performed of the prospectively maintained institutional trauma registry at Massachusetts General Hospital ([MGH] Boston, MA), an urban academic level 1 trauma center. We included all geriatric patients (age ≥ 65 years) admitted to MGH between January 2011 and December 2018 after presenting to the emergency department (ED) within 72 h of a fall resulting in tICH were included. Patients with concomitant, i.e. extracranial injuries with AIS > 2 were excluded. Patients were assigned to cohorts based on pre-injury exposure to anticoagulant medication, resulting in a DOAC-cohort, a VKA-cohort, and a control cohort of patients not using anticoagulant medication. The following data were collected from the trauma registry: age, sex, Charlson comorbidity index (CCI), smoking status, substance abuse, ED vital signs (systolic blood pressure, pulse, temperature, respiratory rate), Glasgow coma scale (GCS), injury severity as measured by injury severity score (ISS) and abbreviated injury scale (AIS) head score, type of ICH, Rotterdam CT score, admission to the intensive care unit (ICU), hospital and ICU length of stay; mechanical ventilation requirement and duration, and discharge disposition [20]. The trauma registry data were enriched with additional variables obtained through manual review of patient records: exposure to anticoagulant medication, specific reversal agent use, admission laboratory values (platelet count, hemoglobin concentration, international normalized ratio [INR]), presence of skull fractures, and neurosurgical interventions.

The outcomes of interest were use of anticoagulant-specific reversal agents (i.e. vitamin K, idarucizumab and andexanet alfa); number and types of neurosurgical interventions; mortality, and discharge to inpatient rehabilitation. The definition of inhospital mortality includes patients discharged to hospice care. Similarly, 3-day mortality includes patients discharged to hospice care on or before hospital day 3. Discharge to inpatient rehabilitation was analyzed only in patients surviving until discharge.

All normally distributed continuous variables are displayed as means with standard deviations. Median and interquartile range notation was used for nonparametric data. Normally distributed continuous variables were compared using one-way ANOVA test and using Kruskall–Wallis test for skewed distributions. Categorical variables are summarized as proportions and compared using Chi^2^-analysis. Missingness of each variable was summarized. Missingness was assumed to be not-at-random, and missing data were imputed using multiple imputation with chained equations. Multivariable logistic regression was performed on the dataset with imputed missing data to assess the independent associated between pre-injury anticoagulant exposure and the use of anticoagulant-specific reversal agents, neurosurgical interventions, discharge to inpatient rehabilitation, 3-day mortality, and inhospital mortality. All variables with a *p* value of less than 0.100 on univariate analysis were included as covariates, with the exception of INR on admission, as it does not reflect the degree of anticoagulation for DOAC-anticoagulated patients, thereby biasing the results toward favorable effects for VKA patients. Only anticoagulated patients were included in the baseline comparison and regression analysis of use of anticoagulant-specific reversal agents.

Considering the large number of covariables studied, collinearity analysis was performed. A mean variance inflation factor (VIF) over five was considered an indicator of collinearity. New multivariable logistic regression models were constructed after sequentially omitting the variable with the strongest collinearity until the mean VIF was below five.

## Results

A total of 1453 patients meeting all inclusion criteria were identified in the trauma registry. Of these, 52 used pre-injury DOACs, 376 used pre-injury VKAs, and 1025 used neither DOAC nor VKA (Table 1). All rates, numbers and scores on univariable analyses are reported as DOAC vs VKA vs control, unless stated otherwise. Data were most frequently missing for ED vital signs, the Rotterdam CT score and admission laboratory values. No significant differences in age or sex were found between groups, but a higher median CCI was reported in the VKA group (4 vs 5 vs 4, *p* < 0.001). The majority of patients were transferred from an outside hospital. No clinically relevant differences were found between groups on presentation, except for median INR, which was highest in the VKA group (1.3 vs 1.8 vs 1.0, *p* < 0.001). No differences between the groups were found for platelet count, hemoglobin and blood alcohol concentration.

**Table 1:** Baseline characteristics of geriatric patients with traumatic intracranial hemorrhage after a fall

Variable	DOAC (*n* = 56)	VKA (*n* = 431)	No OAC (*n* = 1237)	*P* value	% Missing
Median age (IQR)	83 [75–88]	83 [77–87]	81 [74–88]	0.204	0.0
% Female	40.4%	45.5%	51.3%	0.064	0.0
Median CCI (IQR)	4 [4–5]	5 [4–5]	4 [3–5]	** < 0.0005**	0.0
Charlson comorbidty index				**–**	0.0
0–1	0.0%	0.0%	0.1%	**–**	**–**
2–3	23.1%	14.1%	25.6%	**–**	**–**
4	38.5%	34.0	34.4%	**–**	**–**
> 4	38.5%	51.9%	39.9%	**–**	**–**
Transferred from outside hospital	61.5%	61.7%	63.4%	0.823	0.0
Pulse, mean (SD)	83 (18)	81 (19)	80 (16)	0.351	1.7
Systolic blood pressure, mean (SD)	146 (27)	147 (28)	154 (30)	** < 0.005**	1.5
Respiratory rate, mean (SD)	18 (2)	18 (2)	18 (2)	0.607	8.1
Temperature in Celsius, mean (SD)	36.4 (0.5)	36.5 (0.5)	36.5 (0.5)	0.557	17.6
Glasgow coma scale—total, median (IQR)	15 [14–15]	15 [14–15]	15 [14–15]	0.117	1.8
Eye	4 [4–4]	4 [4–4]	4 [4–4]	**–**	**–**
Motor	6 [6–6]	6 [6–6]	6 [6–6]	**–**	**–**
Verbal	5 [4–5]	5 [4–5]	5 [4–5]	**–**	–
TBI severity				0.117	1.7
Mild (GCS 13–15)	94.1%	82.4%	81.4%	–	–
Moderate GCS (8–12)	5.9%	6.0%	6.0%	–	–
Severe (GCS < 8)	0.0%	11.7%	12.5%	–	**–**
INR	1.3 [1.1–1.4]	1.8 [1.3–2.5]	1.0 [1.0–1.1]	** < 0.0005**	19.3
Platelet count in k/uL, median [IQR]	211 (99)	193 (82)	207 (73)	0.047	29.2
Hemoglobin on admission in g/dL, mean (SD)	12.1 (1.9)	11.5 (2.0)	12.2 (1.9)	** < 0.0005**	33.1
% Alcohol intoxication above legal limits*	3.9%	3.5%	4.8%	0.553	0.0

Bold values correspond to a significance level of *p* < 0.05

*DOAC* direct oral anticoagulant, *VKA* vitamin K antagonist, *OAC* oral anticoagulant, *IQR* interquartile range, *CCI* Charlson comorbidity index, *SD* standard deviation, *TBI* traumatic brain injury, *INR* international normalized ratio, *IQR* interquartile range

*Blood alcohol content over 0.08% is considered above legal limits

### Injury pattern

ISS did not differ between the three groups (median ISS: 17 in all groups,  = 0.173; Table 2). The median AIS-head score did not differ significantly between the groups (4 vs 4 vs 4, *p* = 0.079). The majority of patients sustained subdural and/or subarachnoid hemorrhages. Hemorrhagic contusions and intraparenchymal hemorrhage were more frequent in the DOAC group (40.4% *p*vs 25.0% vs 32.7%, *p* = 0.007). The median Rotterdam CT score did not differ significantly between the groups (2 vs 2 vs 2, *p* = 0.281). Skull fractures were most prevalent in the control group (11.5% vs 6.1% vs 18.0%, *p* < 0.0005).

**Table 2:** Injury patterns of geriatric patients with traumatic intracranial hemorrhage after a fall

Variable	DOAC (*n* = 56)	VKA (*n* = 431)	No OAC (*n* = 1237)	*P* value	% Missing
Injury severity score	17 [10–23]	17 [16–25]	17 [15–25]	0.173	0.0
Head abbreviated injury score	4 [3–4]	4 [4–5]	4 [4– 5]	0.079	0.0
ICH Type					
Subdural	63.5%	75.0%	72.1%	0.183	0.0
Epidural	0.0%	1.9%	3.0%	0.234	0.0
Subarachnoid	57.7%	58.2%	58.0%	0.991	0.0
Hemorrhagic contusion and intraparenchymal hemorrhage	40.4%	25.0%	32.7%	**0.007**	0.0
Multiple ICH types	48.1%	46.8%	47.3%	0.978	0.0
Total rotterdam CT score	2 [1–2]	2 [2–2]	2 [2–2]	0.281	26.9
Skull fracture	11.5%	6.1%	18.0%	** < 0.0005**	0.0

*DOAC* direct oral anticoagulant, *VKA* vitamin K antagonist, *OAC* oral anticoagulant, *AIS* abbreviated injury scale, *IQR* interquartile range, *TBI* traumatic brain injury, *ICH* traumatic intracranial hemorrhage, *CT* computed tomography

### Treatment and outcomes

DOAC patients were significantly less likely to receive a specific reversal agent than VKA patients (28.9% vs 60.4%, *p* < 0.001; Table 3). Three DOAC patients received idarucizumab (5.8%) and eight received andexanet alfa (15.4%), whereas 60.4% of VKA patients received vitamin K. Interestingly, seven DOAC patients received off-label vitamin K. DOAC patients underwent neurosurgical interventions significantly more frequently (17.3% vs 13.0% vs 9.5%, *p* = 0.046). More patients in the DOAC group underwent decompressive craniotomy, but this difference was not statistically significant (9.6% vs 7.7% vs 5.1%, *p* = 0.092). No difference was seen in the rate of burr holes and intracranial pressure monitor placement. Extraventricular drain placement was performed significantly more frequently in the DOAC group (5.8% vs 1.3% vs 0.8%, *p* = 0.003). Fewer patients in the DOAC group were admitted to the ICU (39.3% vs 47.1% vs 26.9%, *p* = 0.004), with no difference in duration of ICU stay (median 3 vs 3 vs 3 days, *p* = 0.830). There was no statistically significant difference in mechanical ventilation rates, ventilation days or hospital length of stay. In-hospital mortality or discharge to hospice care was highest in the VKA group and lower in the DOAC and control groups (11.5% vs 25.0% vs 18.2%, *p* = 0.006). This pattern was also seen for mortality or discharge to hospice care at day 3, albeit not statistically significant on univariate analysis (1.9% vs 10.9% vs 8.8%, *p* = 0.088). Fewer patients in the DOAC group were discharged to inpatient rehabilitation than in the VKA and control groups (23.9% vs 44.3% vs 36.8%, *p* = 0.010).

**Table 3:** Treatment and outcomes of geriatric patients with traumatic intracranial hemorrhage after a fall

Variable	DOAC (*n* = 56)	VKA (*n* = 431)	No. OAC (*n* = 1237)	*P* value	% Missing
Anticoagulant-specific reversal agents	28.9%	60.4%	0.0%	** < 0.005**	0.0
Vitamin K	13.5%	60.4%	0.0%	** < 0.005**	0.0
Andexanet alfa	15.4%	0.0%	0.0%	** < 0.005**	0.0
Idarucizumab	5.8%	0.0%	0.0%	** < 0.005**	0.0
Neurosurgical intervention	17.3%	13.0%	9.5%	**0.046**	0.0
Decompressive craniotomy	9.6%	7.7%	5.1%	0.092	0.0
Burr holes	3.9%	4.0%	3.3%	0.825	0.0
Bolt/monitor placement	0.0%	1.9%	2.3%	0.477	0.0
Extraventricular drain placement	5.8%	1.3%	0.8%	**0.003**	0.0
ICU admission	39.3%	47.1%	26.9%	**0.004**	0.0
ICU length of stay (Days)	3 [1–4]	3 [1–6]	3 [1–5]	0.830	0.0
Mechanical ventilation assistance	5.8%	3.8%	5.1%	0.540	0.0
Ventilation duration (days)	2 [1–3]	2 [1–5]	2 [1–4]	0.942	0.0
Total hospital length of stay (Days)	4 [3–8]	5 [3–9]	5 [3–8]	0.148	0.0
In-hospital mortality or hospice	11.5%	25.0%	18.2%	**0.006**	0.0
Mortality at day 3	1.9%	10.9%	8.8%	0.088	0.0
Discharge to inpatient rehabilitation	23.9%	44.3%	36.8%	**0.010**	0.0

*DOAC* direct oral anticoagulant, *VKA* vitamin K antagonist, *OAC* oral anticoagulant, *ICU* intensive care unit, *IQR* interquartile range

### Multivariable logistic regression

Results of the multivariable logistic regression analyses (post-collinearity correction) are presented in Table 4. Pre-injury DOAC use was significantly associated with lower odds of receiving specific reversal agents than pre-injury VKA use [odds ratio (OR) 0.28, 95% confidence interval (CI) 0.15–0.54]. Pre-injury DOAC use, as well as VKA use were independently associated with requiring neurosurgical intervention (DOAC: OR 3.14, 95% CI 1.36–7.28; VKA: OR 1.41, 95% CI 0.94–2.10). Pre-injury DOAC use was not associated with 3-day mortality, nor with inhospital mortality or discharge to hospice care. Pre-injury VKA use was independently associated with higher inhospital mortality or discharge to hospice care (OR 1.62, 95% CI 1.15–2.27). Higher odds of discharge to inpatient rehabilitation were associated with pre-injury VKA use (OR 1.41, 95% CI 1.06–1.87). Sequentially removing the three most strongly correlated covariates until the mean VIF was below five resulted in removal of admission hemoglobin, respiratory rate and platelet count. Post-correction effect directions did not change, and post-correction effect sizes were only marginally different (Table 5).

**Table 4:** Multivariable analyses assessing outcomes after pre-injury coagulant use in geriatric patients with traumatic intracranial hemorrhage after a fall

Variable	Outcome model				
	Specific reversal agent	Neurosurgical intervention	In-hospital mortality or hospice	Day 3 mortality or hospice	Inpatient rehabilitation
Pre-injury anticoagulant	–	–	–		–
DOAC	0.28 (0.15–0.54)	**3.14 (1.36–7.28)**	1.06 (0.42–2.68)	0.58 (0.76–4.40)	0.62 (0.31–1.25)
VKA	1.00 (Reference)	1.41 (0.94–2.10)	**1.62 (1.15–2.27)**	1.43 (0.89–2.31)	**1.41 (1.06–1.87)**
None	–	1.00 (Reference)	1.00 (Reference)	1.00 (Reference)	1.00 (Reference)
Male sex	1.00 (0.67–1.50)	0.78 (0.54–1.13)	0.92 (0.68–1.26)	1.21 (0.79–1.87)	1.17 (0.91–1.49)
CCI (per point)	0.99 (0.85–1.15)	**0.80 (0.69–0.93)**	**1.34 (1.20–1.49)**	**1.24 (1.07–1.44)**	1.02 (0.93–1.12)
TBI severity (Glasgow coma scale)	–	–	–		–
Mild (13–15)	1.00 (Reference)	1.00 (Reference)	1.00 (Reference)	1.00 (Reference)	1.00 (Reference)
Moderate (9–12)	1.40 (0.59–3.34)	1.49 (0.78–2.87)	**3.33 (2.04–5.44)**	**3.17 (1.59–6.34)**	1.43 (0.83–2.44)
Severe (3–8)	0.93 (0.47–1.82)	1.28 (0.80–2.05)	**9.72 (6.48–14.57)**	**12.81 (7.91–20.75)**	1.59 (0.92–2.75)
AIS-head (per point)	1.11 (0.86–1.44)	**5.57 (3.93–7.89)**	**2.60 (2.04–3.31)**	**3.16 (2.16–4.63)**	**1.60 (1.36–1.89)**
Other Tbi	0.94 (0.59–1.51)	**0.63 (0.40–0.99)**	**1.87 (1.33–2.63)**	1.52 (0.95–2.43)	1.17 (0.88–1.56)
Skull fracture	0.78 (0.34–1.76)	1.20 (0.69–2.08)	1.19 (0.77–1.85)	1.65 (0.95–2.88)	0.93 (0.63–1.37)

*DOAC* direct oral anticoagulant, *VKA* vitamin K antagonist, *CCI* Charlson comorbidity index, *TBI* traumatic brain injury, *ISS* injury severity score

Results are post-collinearity correction and are presented as odd ratio (95% Confidence interval)

**Table 5:** Collinearity analysis and corresponding effect per statistical model pre- and post-collinearity correction

Metric	Treatment group	Study outcome				
		Specific reversal agent	Neurosurgical intervention	In-hospital mortality or hospice	Day 3 mortality or hospice	Inpatient rehabilitation
Mean VIF pre-correction		9.53	9.10	9.10	9.10	9.10
OR (95% CI)	DOAC	**0.29 (0.15–0.56)**	**3.14 (1.35–7.29)**	1.04 (0.41–2.64)	0.59 (0.08–4.48)	0.59 (0.29–1.19)
	VKA	1.00 (Reference)	1.40 (0.93–2.10)	**1.53 (1.08–2.15)**	1.44 (0.89–2.33)	**1.35 (1.01–1.80)**
	Control	–	1.00 (Reference)	1.00 (Reference)	1.00 (Reference)	1.00 (Reference)
Mean VIF post-correction		3.72	3.42	3.42	3.42	3.24
OR (95% CI)	DOAC	**0.28 (0.15–0.54)**	**3.12 (1.35–7.25)**	1.06 (0.42–2.68)	0.58 (0.08–4.41)	0.62 (0.31–1.25)
	VKA	1.00 (Reference)	1.40 (0.93–2.09)	**1.62 (1.15–2.27)**	1.44 (0.90–2.31)	**1.40 (1.06–1.87**)
	Control	–	1.00 (Reference)	1.00 (Reference)	1.00 (Reference)	1.00 (Reference)

## Discussion

Treatment of anticoagulated patients and prognosis of intracranial hemorrhage differed significantly depending on pre-injury anticoagulant exposure in geriatric trauma patients with traumatic intracranial hemorrhage after a fall. Pre-injury DOAC users were less likely to receive reversal therapy and more likely to undergo a neurosurgical intervention than patients using VKA or no anticoagulation medication. Pre-injury DOAC use was not associated with mortality or discharge to hospice care, whereas pre-injury use of VKAs was an independent risk factor for mortality.

Clinical outcomes for anticoagulated blunt tICH patients have been the subject of substantial interest: numerous observational cohort studies have compared presentation, management, and outcomes of tICH patients on pre-injury DOACs and VKAs [21–31]. The inhospital mortality rate for DOAC patients found in our study (12%) falls within the range (8–40%) reported in previous studies comparing pre-injury DOACs and VKA in the setting of tICH [21–27]. Comparing our results to the published literature is complicated by widely varying directions of effect and effect sizes between studies, despite comparable baseline characteristics of cohorts of mostly geriatric, comorbid patients presenting with tICH after falls. Differences in patient management, on the other hand, are a likelier cause for differences in outcomes.

First, the rate of DOAC reversal varied, with individual studies reporting between 12 and 40% use of predominantly nonspecific reversal agent use [25, 27, 28, 30, 31]. In our study, 21% of DOAC patients received specific reversal agents, out of 29% receiving reversal at all. Interestingly, all studies that reported the rate of reversal agent use found a significantly lower reversal rate for DOAC patients, while at the same time reporting similar or improved survival rates, comparable to our results. Our results support the hypothesis that DOAC-tICH patients have improved outcomes, despite receiving fewer reversal agents.

Second, rates of neurosurgical intervention in smaller cohorts showed widely differing rates at times favoring pre-injury DOACs and at other times favoring VKAs, while larger, more contemporaneous cohorts reported similar to potentially rates of neurosurgery in for pre-injury DOAC patients [21, 23, 26–28, 31]. The neurosurgical intervention rate for DOAC patients in our study is comparable to that in most recent cohorts (16% vs 10–18%) [27, 28, 31]. It is possible that patient selection for neurosurgical intervention has improved over the years, as providers have become more experienced in managing DOAC-anticoagulation in the trauma setting. In the same spirit, whereas early cohort studies comparing VKA to DOACs reported higher mortality for DOAC patients, more recent studies reported improved survival and higher levels of functioning, indicating that perhaps overall management of DOAC-anticoagulated tICH patients has improved after increasing exposure and experience [27, 28, 30]. Specific temporal trends analyses of pre-injury DOAC and VKA may further explore this hypothesis.

Owing to these differences in patient management, the true association between pre-injury anticoagulation and clinical outcomes is difficult to determine. The most reliable information on outcomes of anticoagulated tICH patients could come from subset analyses from randomized controlled trials comparing efficacy and safety of chronic oral anticoagulant use. In two studies comparing DOACs to VKAs (RE-LY and ROCKET-AF), however, sample sizes were too small to determine a survival benefit of either type in the setting of tICH [32, 33].

Alternatively, studies using national databases may diminish the impact of heterogeneity between hospitals, as well as sample size issues. While the association between pre-injury anticoagulation, treatment and outcomes has been studied in the general trauma population, analysis of the tICH subpopulation may add further information [34].

### Limitations

This study is limited by several factors arising from its retrospective design. First, we were not able to report ICH progression rate at a fixed timepoint. While ICH progression is sometimes preferred over mortality as an outcome, especially in studies of treatment effects in the early phase of tICH management, this study aimed to describe management and prognosis, and therefore discharge to inpatient rehabilitation and mortality were deemed appropriate outcomes. Secondly, retrospective data collection impeded collection of the modified Rankin scale, which is commonly used in studies to assess general neurological functioning after stroke and intracranial hemorrhage. We did also not account for the degree of anticoagulation, as anti-FXa assays were not used, nor were such assays validated during the study period. Moreover, the use of non-anticoagulant-specific reversal agents, e.g. 3 or 4 factor prothrombin complex concentrate, was not collected in this study. The DOAC group was smaller than the VKA cohort. Imbalance in group sizes reduces the statistical power of tests performed, resulting in lower chances of detecting significant differences between groups. Despite this limitation, reported differences in reversal agent use, neurosurgical interventions, and mortality or hospice care were statistically significant. Sensitivity analysis demonstrated a limited impact of collinearity, as evidenced by the effect sizes not changing significantly pre- and post-collinearity correction.

## Conclusion

In patients with tICH after falls, pre-injury DOAC was associated with similar rates of mortality and discharge to inpatient rehabilitation, whereas pre-injury VKA exposure was associated with higher inhospital mortality and more frequent discharge to inpatient rehabilitation. Vigilance remains warranted in the treatment of DOAC-anticoagulated trauma patients, as differences in treatment allude to various risk profiles. Future research should focus on risk assessment and stratification of DOAC-exposed trauma patients.
