# Correction to: Treatment and outcomes of anticoagulated geriatric trauma patients with traumatic intracranial hemorrhage after falls

**DOI:** 10.1007/s00068-022-02024-8

**Published:** 2022-06-14

**Authors:** Charlie J. Nederpelt, Leon Naar, Karien Meier, Suzanne F. M. van Wijck, Pieta Krijnen, George C. Velmahos, Haytham M. A. Kaafarani, Martin G. Rosenthal, Inger B. Schipper

**Affiliations:** 1 Department of Trauma Surgery, Leiden University Medical Center, Leiden, The Netherlands; 2 Division of Trauma, Emergency Surgery and Surgical Critical Care, Massachusetts General Hospital, Boston, MA United States; 3 Department of Trauma Surgery, Erasmus Medical Center, Rotterdam, The Netherlands

## Correction to: European Journal of Trauma and Emergency Surgery 10.1007/s00068-022-01938-7

In this article the author names Charlie J. Nederpelt, Suzanne F. M. van Wijck, George C. Velmahos, Haytham M. A. Kaafarani, Martin G. Rosenthal, Inger B. Schipper was incorrectly written as Charlie Nederpelt, Suzanne van Wijck, George Velmahos, Haytham Kaafarani, Martin Rosenthal, Inger Schipper.

The original article has been corrected.

